# Association of cannabis with glutamatergic levels in patients with early psychosis: Evidence for altered volume striatal glutamate relationships in patients with a history of cannabis use in early psychosis

**DOI:** 10.1038/s41398-020-0790-1

**Published:** 2020-04-21

**Authors:** Musa Sami, Amanda Worker, Marco Colizzi, Luciano Annibale, Debasis Das, Marlene Kelbrick, Savitha Eranti, Tracy Collier, Chidimma Onyejiaka, Aisling O’Neill, David Lythgoe, Philip McGuire, Steve C. R. Williams, Matthew J. Kempton, Sagnik Bhattacharyya, Collaborators, Praveen Macherla, Athanasios Prountzos, Rachel Kitts, Loredana Vasicuro, Zohra Taousi, Fatma Tekfi

**Affiliations:** 1 Institute of Psychiatry, Psychology and Neurosciences, King’s College London, London, UK; 2 Institute of Mental Health, Nottingham University, Nottingham, UK; 3 Section of Psychiatry, Department of Neurosciences, Biomedicine and Movement Sciences, University of Verona, Verona, Italy; 4 Leicestershire Partnership NHS Trust, Thurmaston, UK; 5 Northamptonshire Healthcare NHS Foundation Trust, Kettering, UK; 6 East London Foundation Trust, London, UK; 7 South West London and St George’s Mental Health NHS Trust, London, UK; 8 Surrey & Borders Partnership NHS Foundation Trust, Redhill, UK; 9 Hertfordshire Partnership University NHS Foundation Trust, St Albans, UK

**Keywords:** Molecular neuroscience, Schizophrenia

## Abstract

The associative striatum, an established substrate in psychosis, receives widespread glutamatergic projections. We sought to see if glutamatergic indices are altered between early psychosis patients with and without a history of cannabis use and characterise the relationship to grey matter. 92 participants were scanned: Early Psychosis with a history of cannabis use (EPC = 29); Early Psychosis with minimal cannabis use (EPMC = 25); Controls with a history of cannabis use (HCC = 16) and Controls with minimal use (HCMC = 22). Whole brain T1 weighted MR images and localised proton MR spectra were acquired from head of caudate, anterior cingulate and hippocampus. We examined relationships in regions with known high cannabinoid 1 receptor (CB1R) expression (grey matter, cortex, hippocampus, amygdala) and low expression (white matter, ventricles, brainstem) to caudate Glutamine+Glutamate (Glx). Patients were well matched in symptoms, function and medication. There was no significant group difference in Glx in any region. In EPC grey matter volume explained 31.9% of the variance of caudate Glx (*p* = 0.003) and amygdala volume explained 36.9% (*p* = 0.001) of caudate Glx. There was no significant relationship in EPMC. The EPC vs EPMC interaction was significant (*p* = 0.042). There was no such relationship in control regions. These results are the first to demonstrate association of grey matter volume and striatal glutamate in the EPC group. This may suggest a history of cannabis use leads to a conformational change in distal CB1 rich grey matter regions to influence striatal glutamatergic levels or that such connectivity predisposes to heavy cannabis use.

## Introduction

A third of patients presenting with First Episode Psychosis use cannabis regularly^1^. This dual diagnosis cohort has a worse clinical outcome for days in hospital, relapses and long term health^2–5^ not accounted for by potential confounders such as alcohol, other drug use, adherence to treatment and severity of illness at onset^6^, differences in genetic make-up or premorbid environment^7^. Cessation of cannabis use in patients with psychosis improves outcome^3^, but is difficult to achieve^8^. Comorbid cannabis use is associated with a higher incidence of treatment failure in psychosis^2^. While there is some evidence that clozapine reduces cannabis craving and use, there are no clear indications for superiority of any antipsychotic^9^. There are no current effective pharmacological or psychological interventions that can mitigate harm from cannabis use in people with psychosis^10,11^. Hence, there is a pressing need to understand the neurobiological alterations in the dual diagnosis group, which may inform the development of more effective treatments.

Positive psychotic symptoms may be underpinned by increased presynaptic dopamine synthesis capacity^12,13^. However there is limited evidence that cannabinoid administration in humans increases dopaminergic signalling^14^. Positron Emission Tomography (PET) studies in healthy volunteers with psychotic symptoms induced by Δ-9-tetrahydrocannabinol (THC) administration showed limited displacement of D2 receptor radioligand binding^15–17^. In cannabis users there is evidence of a blunting, rather than increase of dopaminergic responses with no relation between dopamine synthesis capacity and induction of psychotic symptoms^18^. If cannabis use does not increase striatal dopamine levels an alternative mechanism must be sought to explain the psychosis-cannabis association.

Psychosis also appears to be related to alterations in brain glutamate function^19,20^. Glutamatergic perturbations can be indexed using proton Magnetic Resonance Spectroscopy (MRS), with abnormalities observed in patients versus controls^21^. There are compelling reasons to investigate the glutamate system within the context of cannabis use in psychosis. Preclinical evidence suggests an extensive disruption of the glutamate function in the context of cannabinoid exposure^22^, whereas chronic cannabis use appears to decrease glutamate in otherwise healthy individuals^22^. The Cannabis Receptor 1 (CB1R), the binding site for Δ-9-THC, predominantly found in axon terminals of the grey matter, is amongst the most widely distributed G-Protein Coupled Receptor in the brain with concentrations 10-50 times that of other neurotransmitters^23^. There are widespread pyramidal projections from regions with high CB1 receptor expression with the associative striatum at the head of caudate where glutamate is the primary neurotransmitter. At the synapse CB1R has been proposed to be involved in post-synaptic hypofunction of NMDA Receptor via receptor internalisation and CB1R has been noted to interact with glutamate NMDA-Receptors to modulate long-distance neural oscillatory activity^24,25^.

To date, only one study has examined glutamate levels in patients with and without a history of cannabis use: showing a decrease of Glx in the prefrontal cortex in cannabis-using versus non-using patients with psychosis^26^. However, this study neither examined changes in other brain regions of interest nor did they include a cannabis using, otherwise healthy control group.

In the context of dual diagnosis the striatal glutamate has become a site of particular interest. Striatal glutamatergic levels, as typically indexed by glutamate+glutamine (Glx) from Magnetic Resonance Spectroscopy, have been shown to be elevated in patients during first presentation of psychosis as compared to controls and a high risk group versus controls^27,28^. Furthermore, two recent double-bind randomised controlled studies have shown an increase in striatal glutamate after administration of Δ-9-THC in healthy volunteers^29,30^ and in one study this has been shown to be associated with a perturbation of cortico-striatal connectivity^29^.

Therefore, in the present study we investigated whether glutamatergic indices were altered in early psychosis patients with and without a history of cannabis use and in a comparable group of otherwise healthy individuals with and without a history of cannabis use. We hypothesized, on the basis of pilot data that relative to patients without a history of cannabis use, patients with a history of cannabis use would show increased glutamatergic indices in brain regions implicated in psychosis (anterior cingulate cortex^31,32^, hippocampus^33^, and caudate^34^) and that levels in patients would be greater than in controls. Given the widespread distribution of the CB1R throughout the grey matter^35^ and established pyramidal projections to the associative striatum, we further hypothesized that regions rich in CB1R expression (total grey matter, amygdala, hippocampus, cortex) would be associated with caudate Glx in patients with a history of cannabis use. As in previous studies the primary metabolite of interest in all regions was the composite Glx peak (glutamate + glutamine) as a marker of glutamatergic function, as it likely reflects glutamate levels which are typically 5–6 times higher than glutamine^36^, and has been shown to be increased in the head of caudate in the two RCTs of acute challenge of Δ-9-THC^29,30^ and in First Episode patients^27^. Results for the Glutamate (Glu) metabolite are presented as secondary analysis.

## Methods

### Sample

In the Effect of Cannabis in Psychosis (EfCiP) study (London-Stanmore REC 17/LO/0577) we collected data from four groups: patients with Early Psychosis with a history of cannabis use (EPC), patients with early psychosis with a minimal exposure to cannabis use (EPMC), healthy control participants with a history of cannabis use (HCC) and healthy control participants with minimal exposure to cannabis use (HCMC). Informed consent was obtained from all participants. Patients were referred from Early Intervention in Psychosis services from 16 National Health Service trusts in England. Controls were identified from a register of healthy volunteers and individuals expressing interest in cannabis research in an online survey^37^. Participants were aged 18–38 years and patients were clinically stable on treatment. We excluded individuals with a diagnosis of organic psychosis. Infrequent experimentation with cannabis is common in the general population and therefore non-cannabis using participants were defined as those having used cannabis ≤20 times in their life. This cut-off for significant use is consistent with previous studies^38,39^. All minimal cannabis users (EPMC, HCMC) reported use a few times a year, only once or twice or not at all; whereas all cannabis users (EPC, HCC) reported use at least a few times each month, more than weekly or daily (for full breakdown see Supplementary Table 1). Final group allocation was made after Structured Clinical Interview for DSM-IV (SCID) interview on the study day.

Of 103 participants recruited, one HCC was excluded due to cannabis intoxication, one HCMC was excluded due to prolactinoma, two participants (EPMC) experienced claustrophobia and were unable to have an MRI scan, six participants (3 EPC, 3 EPMC) were not able to have MRI due to contraindications to MRI scanning. One patient had a first psychotic episode aged 12, 20 years and was excluded. Data were available for 92 participants in the study: EPC: *n* = 29, EPMC: *n* = 25; HCC: *n* = 16; HCMC: *n* = 22.

Early psychosis was defined as presentation to secondary mental health services with psychosis within the last 5 years. This patient was currently under treatment of an early intervention in psychosis team within the first 5 years and was retained in the study. One HCC suffered from Generalised Anxiety Disorder on no treatment and one HCC had Obsessive Compulsive Disorder 8 years prior, currently in remission maintained on low dose sertraline (50 mg).

### Power

The study was powered prospectively based on effect-size (Cohen’s *d* = 1.052) estimated from interim data from a separate study in our laboratory comparing hippocampal glutamate in healthy cannabis users with non-users (*n* = 19). A sample size of *n* = 16 per group was required to detect differences between the EPC and EPMC groups on 1H-MRS glutamate with an alpha (α) of 0.05 at 80% power. This was consistent with the only previous study in patients in this area, subsequently published, which found a significant difference in 17 cannabis users, 18 non-cannabis users and 35 controls^26^.

### Clinical measures

Measures undertaken were the modified Cannabis Experiences Questionnaire we have used before^6^, Timeline Follow Back (TLFB) questionnaire^40^, Alcohol Use Disorders Identification Test (AUDIT) score for alcohol use in the preceding year^41^ and Fagerstrom Test for Nicotine Dependence^42^. National Adult Reading Test (NART) was applied for Intelligence Quotient estimation based on a recently re-standardised calculation in British adults^43^. Patients with diagnosis of mild Learning Disability (2 EPC, 2 EPMC) were assigned an estimated IQ of 65. All participants underwent Positive and Negative Syndrome Scale (PANSS) and Structured Clinical Interview for DSM-IV (SCID) interview (modules for mood, psychosis and substance use as appropriate from the SCID screening interview) to establish diagnosis and group allocation by experienced raters alongside rating of Global Assessment of Functioning (GAF). Participants underwent Urine Drug Sample (UDS) for recent drug use assessment.

### Participants

were asked not to use cannabis or alcohol from the day before scanning and not to drink coffee on the day prior to the scan. Tobacco use was allowed to avoid withdrawal effects. Participants were asked to abstain from cigarettes from one hour before the scan however two participants (EPC) had cigarettes 15 min before the scan. Exclusion of their data in a sensitivity analysis showed no difference in the main results.

### Data acquisition

Data was acquired using a 32-channel Nova head coil on a General Electric (Chicago, IL, USA) 3-Tesla MR750 system. After a 3-plane localizer for orientation and an ASSET calibration, volumetric T1 weighted MR images were acquired using Sagittal ADNI Go Inversion Recovery Spoiled Gradient Echo (IR-SPGR) sequence with 196 1.2 mm thick slices were acquired with an in-plane matrix size of 256 × 256 (1.05 mm × 1.05 mm) (TR/TI/TE 7.312 ms/400 ms/3.016 ms, flip angle: 11°).The field of view was placed to avoid nose wrap. Total acquisition time was 5 min 37 s.

MR Spectroscopy was undertaken using Point RESolved Spectroscopy (PRESS) in three regions: a 2 × 2 × 2 cm^3^ voxel in the anterior cingulate and head of caudate and a 2 × 2 × 1.5 cm^3^ voxel in the hippocampus. Partial volume correction was applied to all metabolites. For further details of signal acquisition and partial volume correction see Supplementary methods and sFig. 1.

#### Volume

T1 weighted MR images were processed using FreeSurfer 6.0 Massachusetts General Hospital, Harvard Medical School; http://surfer.nmr.mgh.harvard.edu). The standard automated pipeline was applied using *recon-all*. After motion correction the original volume was registered to MNI305 atlas^44^, intensity normalisation was undertaken and skull stripping was performed. Subcortical structures were segmented and labelled by registering images to the Freesurfer average atlas. The following volumetric indices were extracted: total cerebral grey matter (hereafter ‘grey matter’), total cerebral white matter (hereafter ‘white matter’) and total ventricular volume (sum of all ventricles, CSF and choroid plexus as measured by FreeSurfer, hereafter ‘CSF’) as well as volumes for left hippocampus, left amygdala, left cortex and brainstem.

### Quality control

#### Volumes

All structural images were visually inspected for motion and MRI artifacts. After processing steps and further visual inspection, manual edits were made to one scan, no further edits were made and it was not necessary to exclude any data.

#### MRS

Cramer-Rao lower bounds of ≥20% were excluded from MRS analysis as these have been noted to have low reliability. Cramer-Rao lower bounds of remaining scans were checked to ensure there were no significant differences between groups (*p* > 0.45, all regions). For quality parameters see Supplementary Table 2.

### Statistics

Demographic and MRI volume data were compared across groups using ANOVA tests for continuous measures and chi-squared tests or Fisher’s exact test (where individual categories were ≤5) for categorical data. Because the primary comparison of interest was EPC vs EPMC all tests were also run for EPC vs EPMC using *t*-tests and chi squared as appropriate.

As primary dependent variables: partial volume corrected anterior cingulate, hippocampus and caudate Glx levels were checked for normality using Shapiro-Wilks test. ACC and caudate Glx were normally distributed; we undertook logarithmic transformation for Hippocampal Glx to correct positive skew.

To determine whether there was a group difference we ran MANOVA model by entering patient status and cannabis use as the independent variable, Glx levels for the three regions as the dependent variables with follow-up tests if significant. In a further sensitivity analysis we adjusted the model (MANCOVA) covarying for age and sex. To test specific hypotheses of differences between (i) EPC vs EPMC and (ii) all patients and all controls we undertook t-tests in each region. We set significance level at *p* < 0.05.

To examine this further we undertook Pearson’s correlations on total grey matter volume and caudate Glx by group. We also ran correlations in control regions where CB1 receptors are known to be low (white matter) or absent (CSF). P value was Bonferroni-corrected to account for multiple comparisons and groups (*p* < (0.05 ÷ 12) = 0.0042).

To examine the relationship between caudate glutamatergic levels and specific grey matter regions known to have high density of CB1 receptors and projections to the caudate we undertook Pearson’s correlations on left cortex, left hippocampus and left amygdala and caudate Glx by group. We also included brain stem volume as a control region where CB1 receptors are know to be low. *p*-Value was Bonferroni-corrected to account for multiple comparisons and groups (*p* < (0.05 ÷ 16) = 0.0031).

In sensitivity analyses we repeated these tests in grey matter regions to ensure our results were robust to variations in the following parameters: (i) using Glu instead of Glx as the metabolite of interest; (ii) using alternative methods of partial volume correction and (iii) using right sided instead of left sided structures. As exploratory sensitivity analyses we set *p* < 0.05. See Supplementary Tables 3 and 4 for the full results.

To ensure that the relationship between grey matter and caudate Glx in cannabis using patients was not accounted for covariates we ran regression model in the EPC group with caudate Glx as the dependent variables and grey matter, age, sex, Fagerstrom (for smoking) and AUDIT (for alcohol) scores as predictors in the model.

In further exploratory analysis we investigated if Glx levels in any region (hippocampus, amygdala, caudate) were correlated with clinical measures (PANSS, GAF, and Chlorpromazine equivalents) in both patient groups.

Statistics were undertaken in SPSS version 25 (IBM, Armonk, NY, USA).

## Results

### Patient demographics

Patients (EPC vs EPMC) were well matched across clinical parameters including PANSS, GAF and antipsychotic dose in terms of chlorpromazine equivalents (see Table 1). Cannabis users had higher AUDIT and Fagerstrom scores than participants. As expected control participants had higher estimated IQ scores than patients but there was no difference between cannabis using and non-using patients. There were no significant differences in cannabis using parameters between EPC and HCC: average age of first use of cannabis was 16.07 years (s.d. 2.51) for EPC and 16.00 years (s.d. 3.56) for HCC (*p* = 0.92); time to use an eighth of an ounce of cannabis (3.5 grams) was 9.56 days (s.d. 11.36) for EPC and 7.96 (s.d. 9.28) days for HCC (*p* = 0.68) and 12/29 (41%) EPC participants tested positively on Urine Drug Sampling versus 8/16 (50%) for HCC (*p* = 0.547).

**Table 1:** Patient demographics.

	EPC	EPMC	HCC	HCMC	*p*-Value
*n*	29	25	16	22	
Sex	23/29 (79%)	16/25 (64%)	10/16 (63%)	11/22 (50%)	All groups: 0.182
					EPC vs EPNC: 0.210
Age	25.57 (3.89)	26.45 (5.04)	27.11 (5.95)	28.16 (5.29)	All groups: 0.315
					EPC vs EPNC: 0.476
Age at first presentation	23.56 (4.06)	24.10 (5.00)	—	—	EPC vs EPNC: 0.565
AUDIT	8.79 (5.30)	3.40 (4.92)	7.75 (6.43)	3.59 (2.99)	**All groups: <0.001**
					**EPC vs EPNC: 0.001**
Fagerstrom	2.59 (2.44)	0.64 (1.52)	0.75 (1.73)	0	**All groups: <0.001**
					**EPC vs EPNC: 0.001**
Proportion SSD	22/29 (76%)	19/25 (76%)	none	none	EPC vs EPNC: 0.991
Diagnosis					EPC vs EPNC: 0.459
Schizophrenia	11 (38%)	12 (44%)			
Schizoaffective	8 (28%)	6 (24%)			
Schizophreniform	3 (10%)	2 (8%)			
Bipolar	2 (7%)	2 (8%)			
Psychotic depression	1 (3%)	0			
Brief psychotic D	1(3%)	2 (8%)			
Psychosis NOS	0	2 (8%)			
Substance induced	3 (10%)	0			
Addiction comorbidity (lifetime)*					**All groups: <0.001EPC vs EPNC: 0.001**
Cbs Dep	18 (62%)	0	6 (38%)	0	
Cbs abuse	5 (17%)	0	2 (13%)	0	
ETOH Dep	1 (3%)	1 (4%)	1 (6%)	0	
ETOH abuse	4 (14%)	1 (4%)	2 (13%)	0	
Other Dep	2(7%)	1 (4%)	1 (6%)	0	
None	5 (17%)	22 (88%)	7 (44%)	22 (100%)	
PANSS					EPC vs EPNC: 0.526
Positive symptoms	12.17 (5.39)	11.24 (5.31)			EPC vs EPNC: 0.806
Negative symptoms	13.93 (7.23)	14.40 (6.59)			EPC vs EPNC: 0.905
General	27.14 (9.16)	26.84 (9.04)			**All groups: <0.001**
Total	53.24 (18.49)	52.48 (17.46)	34.56 (5.38)	31.05 (2.13)	EPC vs EPNC: 0.878
GAF	70.24 (8.98)	73.16 (11.21)	89.25 (4.93)	93.32 (2.64)	**All groups: <0.001**
					EPC vs EPNC: 0.293
CPZ equivalents	189.57 (174.15)	195.54 (172.43)	none	none	EPC vs EPNC: 0.9
Antipsychotic Type					EPC vs EPNC: 0.801
Atypical Oral	14 (48%)	11 (44%)			
Typical	0	0			
Clozapine	1 (3%)	1 (4%)			
Depot (all atypical)	4 (15%)	6 (24%)			
None	10 (34%)	7 (28%)			
Intelligence quotient	100.46 (12.73)	99.28 (14.59)	110.28 (6.93)	110.23 (7.97)	**All groups: 0.002**
					EPC vs EPNC: 0.768

Data presented in cells proportions for discrete data; means (standard deviations) for continuous data. *p*-Values are reported for omnibus tests (Chi squared, Fisher’s exact where numbers in categories ≤5, ANOVA) for all groups: group-wise comparisons reported as appropriate (Chi squared, *t*-tests). *EPC* early psychosis with cannabis use, *EPMC* early psychosis with minimal cannabis use, *HCC* healthy controls with cannabis use, *HCMC* healthy controls with minimal cannabis use, *n* number of participants, SSD schizophrenia spectrum disorder, *CPZ* chlorpromazine. *Excludes nicotine (see Fagerstrom). Bold typeface indicates significance *p* < 0.05.

### Volume

There were no significant differences between EPC vs EPMC for all volumes of regions and tissue classes of interest (*p* > 0.16). There was a group effect of white matter such that HCC had increased white matter compared to HCMC and EPMC (see Table 2). When corrected for intracranial volume there were no significant differences between all groups for all regions and tissue classes of interest both for all groups (*p* > 0.1) and for EPC vs EPMC (*p* > 0.1).

**Table 2:** Volume relationships between groups (mls).

	EPC	EPMC	HCC	HCMC	*p*-Value
Grey matter	566.3 (61.3)	545.2 (47.5)	579.8 (61.3)	541.7 (40.8)	All group: 0.085
					EPC vs EPMC: 0.167
White matter	456.0 (57.1)	447.9 (48.1)	495.7 (59.8)	437.7 (51.6)	**All group: 0.011**
					**HCC vs HCMC: 0.003**
					**EPMC vs HCMC: 0.033**
					EPC vs EPMC: 0.575
CSF	18.18 (6.7)	19.2 (9.5)	21.5 (9.0)	16.9 (4.9)	All group: 0.319
					EPC vs EPMC: 0.653
L Caudate	3.51 (0.4)	3.4 (0.4)	3.7 (0.5)	3.4 (0.5)	All group: 0.205
					EPC vs EPMC: 0.182
L Hippocampus	4.14 (0.4)	4.0 (0.4)	4.3 (0.4)	4.1 (0.3)	All group: 0.187
					EPC vs EPMC: 0.305
L Amygdala	1.60 (0.2)	1.7 (0.2)	1.7 (0.3)	1.7 (0.3)	All group: 0.274
					EPC vs EPMC: 0.157
L Cortex	252.0 (27.8)	242.8 (21.8)	258.1 (27.5)	241.7 (18.5)	All group: 0.110
					EPC vs EPMC: 0.187
Brainstem	19.0 (2.3)	19.6 (2.0)	20.0 (2.7)	19.0 (1.8)	All group: 0.421
					EPC vs EPMC: 0.360
Intracranial volume	1363.3 (138.2)	1364.6 (176.3)	1459.9 (135.8)	1359.0 (136.4)	All groups: 0.136
					EPC vs EPMC: 0.975

All measures in cm^3^. Mean values given (standard deviations in brackets). *EPC* early psychosis with cannabis use, *EPNC* early psychosis with minimal cannabis use, *HCC* healthy controls with cannabis Use, *HCNC* healthy controls with minimal cannabis use. Bold typeface indicates significance *p* < 0.05.

### Glutamate levels

There was no significant group effect for patients or cannabis users in anterior cingulate, hippocampus, and caudate Glx and no significant patient x cannabis use interaction, both in adjusted and unadjusted models (*p* > 0.26).

**Fig. 1:**
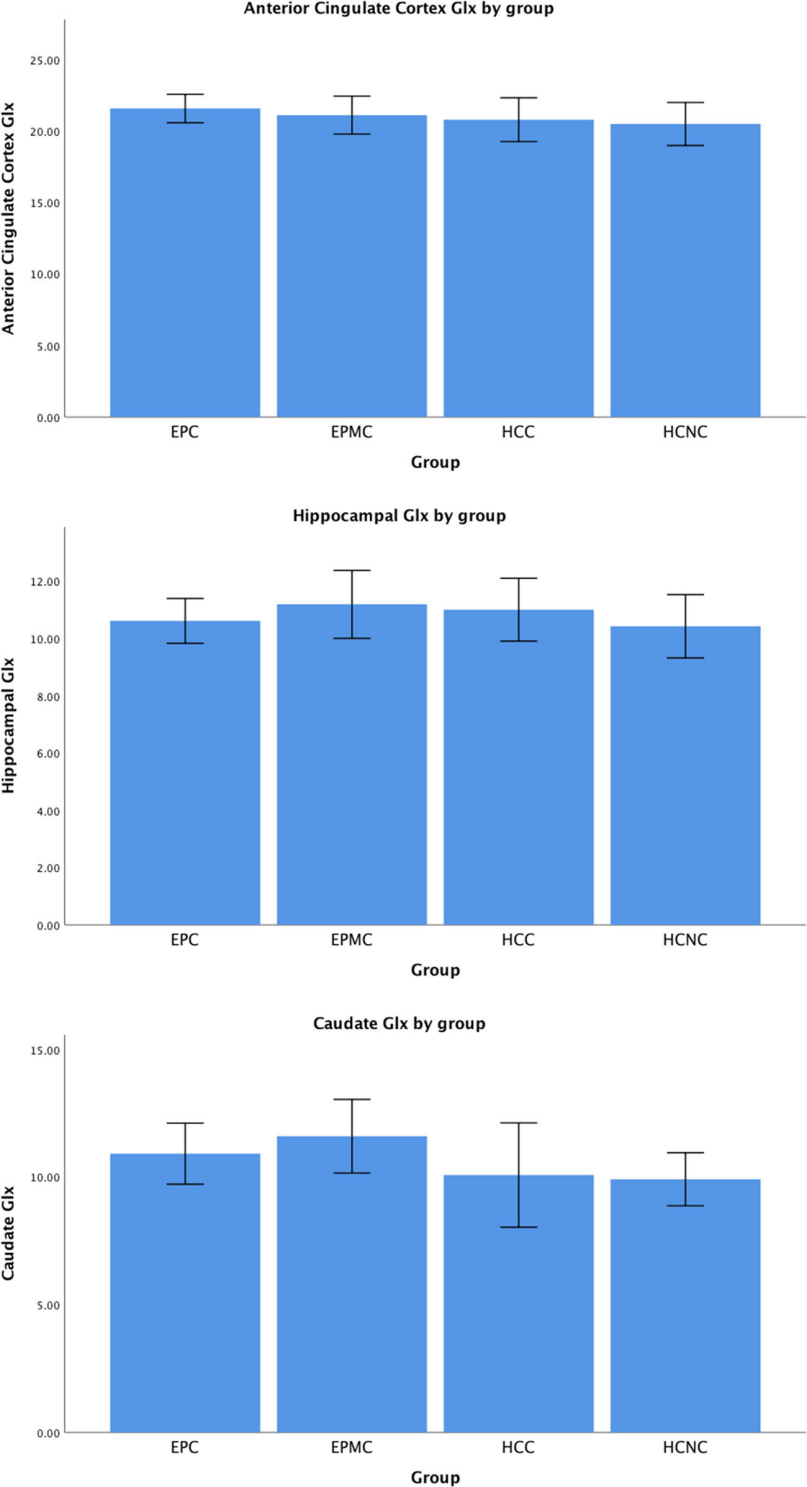
Glx levels by regions across groups. EPC: early psychosis with cannabis use; EPMC: early psychosis without cannabis use; HCC: healthy controls with cannabis use; HCMC: healthy controls without cannabis Use. Glx levels are partial volume corrected. Error bars are 95% confidence Intervals.

To test specific hypotheses we compared (i) EPC vs EPMC and (ii) all patients versus all controls. There were no significant difference for either comparison in any region. Caudate glutamate was elevated in EPMC compared to EPC (*p* = 0.449). There was a trend level increase for Caudate Glx in all patients compared to all controls (*p* = 0.066) but this was not significant and remained at trend level when accounting for different methods of partial volume correction (CSF only: *p* = 0063, no correction: *p* = 0.54). Trend level significance did not remain when Glu was used instead of Glx (*p* > 0.2). Glx levels by group are shown in Fig. 1.

In exploratory analysis of metabolite levels there was no significant group difference in any of the major metabolites (Creatine; Glutamate; myo-inositol; N-acetylaspartate; glycerophosphocholine+phosphocholine) with acceptable Cramer-Rao Lower Bound thresholds in any of the three voxels (*p* > 0.14). Full details are reported in Supplementary Table 5.

### Volume relationship with grey matter

**Fig. 2:**
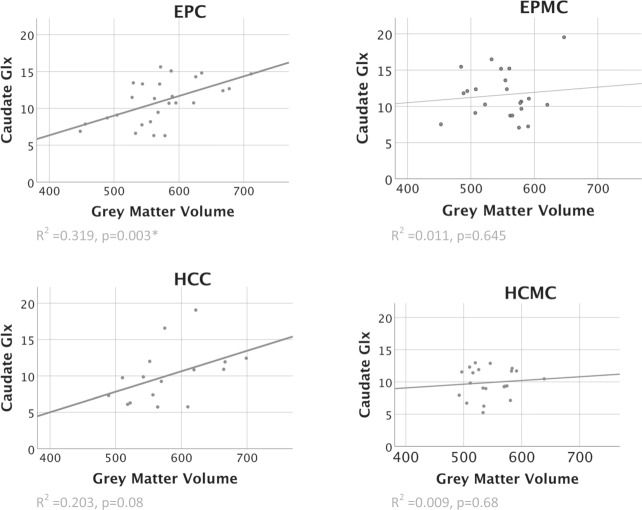
Grey matter volume by caudate Glx across groups. Grey matter measures in cm^3^. EPC: early psychosis with cannabis use; EPMC: early psychosis without cannabis use; HCC: healthy controls with cannabis use; HCMC: healthy controls without cannabis use.

Total grey matter volume was associated with caudate Glx in cannabis-using patients but not in other groups (EPC: *r* = 0.565, *p* = 0.003; see Fig. 2). This met the significance threshold for EPC after multiple comparison correction. No such correlation was seen in other groups (see Fig. 2). The positive correlation seen in the grey matter caudate Glx relationship in EPC was significantly different from the correlation in the EPMC (one-tailed Fisher r-to-z, *z* = 1.72, *p* = 0.042). In control tissue regions there was no relationship in EPC for white matter (*r* = 0.359, *p* = 0.072) or CSF with caudate Glx (*r* = 0.207, *p* = 0.31).

In EPC a relationship between caudate Glx and volume was shown in amygdala (*r* = 0.607, *p* = 0.001) with a trend level in hippocampus (*r* = 0.520, *p* = 0.006) and left cortex (*r* = 0.526, *p* = 0.006), but not brainstem (*r* = 0.339, *p* = 0.091) after multiple comparison correction. There were no other significant findings for relationship between caudate Glx and tissue class or region in any other groups.

Regression analyses showed grey matter and amygdala volume to be a significant predictor of caudate Glx in patients with a history of cannabis use (EPC) after co-varying for AUDIT, Fagerstrom, age and sex (see Table 3).

**Table 3:** Regression models for caudate glx in EPC.

	Beta	*t*	Sig.
(a) Grey matter volume as a predictor of caudate Glx			
Grey matter volume	0.617	2.658	**0.015**
Age	0.169	0.807	0.429
AUDIT score	0.152	0.733	0.472
Fagerstrom score	0.125	0.639	0.53
Sex	−0.079	−0.329	0.746
(b) Amygdala volume as a predictor of caudate Glx			
Left amygdala volume	0.647	2.794	**0.011**
AUDIT score	0.083	0.407	0.688
Age	0.054	0.28	0.782
Fagerstrom score	0.047	0.255	0.802
Sex	0.022	0.086	0.932

Bold typeface indicates significance *p* < 0.05.

### Association of caudate Glx with clinical measures

There was no significant relationship of either patient group for Glx in any region with PANSS, GAF or Chlorpromazine equivalents.

## Discussion

In three brain regions not previously studied we measured glutamatergic indices using an established biomarker (Glx) in psychosis^21^. This is the largest study to date to examine glutamatergic indices in early psychosis patients with and without a history of cannabis use. We found no significant difference between the patient groups and healthy control participants in total Glx across three different regions implicated in psychosis.

A longitudinal study and meta-analytic evidence suggest that increased Glx levels may be a state marker seen in unmedicated patients with psychosis or early stages of illness but not chronic illness or patients on treatment^21,27^. Our patients were a clinically stable, treated outpatient sample, with mean PANSS scores in the mild-moderate range, exposure to antipsychotic treatment or clinical stability may have normalised the glutamatergic alteration^21,45^. Nonetheless a previous study found reductions in glutamate levels in the prefrontal cortex between cannabis and non-cannabis using patients with early psychosis in a similarly treated sample^26^. In a larger sample and across three different regions in a study designed for this purpose, we do not replicate this finding. Possible reasons for this include the use of different regions of interest, differences in scanning parameters and data acquisition, differences in partial volume correction techniques and different cannabis use patterns amongst samples.

This study is, however, the first to demonstrate an association of grey matter volume and striatal glutamate in patients with a history of cannabis use. We found a strong positive relationship between total grey matter volume and caudate Glx levels in cannabis using patients: with grey matter volume explaining 31.9% of the variance of caudate Glx and amygdala volume explaining 36.9% of the variance of caudate Glx.

The CB1R is one of the most widely distributed G Protein Coupled Receptors in the brain, with higher expression in grey matter regions than white matter^46^. Although CB1R density is high in the corpus striatum and ventral striatum, in vitro evidence suggests these are not the site of action for striatal dopamine release^47^. Of interest our findings are demonstrated in the EPC group (*n* = 26), and are at a trend level in the HCC group (*n* = 16). We had preferentially recruited patients in this study but a sample size of 26 in HCC would be powered at 80%, alpha 0.05 for the correlation coefficient we detect. It may be that this association is a cannabis specific effect rather than limited to EPC alone.

Given the cross-sectional nature of the study and the uncertain nature of whether MRS indexes neuronal or metabolic glutamate pools interpretation of these results is by necessity speculative. Our results may suggest that widespread distal projections to the caudate from distributed grey matter regions are implicated in caudate glutamate signalling in patients with a history of cannabis use. In such a model chronic cannabis use leads to a conformational change in distal areas in the brain, particularly regions with high CB1R expression, which influence striatal glutamatergic levels through pyramidal projections to the associative striatum. CB1Rs are found in axonal terminals in the grey matter of glutamatergic terminals and GABAergic interneurons^48^ and activation can lead to both excitation or inhibition^25,48^. There is evidence that chronic cannabis use downregulates CB1R availability across the cortex and other grey matter regions in at least a partially reversible manner^49,50^ and is also involved in desensitization of CB1R activity^22,51^. Perturbed endocannabinoid signalling in chronic cannabis use may also be induced by widespread reductions in Fatty Acid Amide Hydrolase indicating perturbation in endocannabinoid signalling^52,53^, NMDA receptor internalisation^35^, or changes in long term depression and potentiation^54^.

An alternative explanation, which is not mutually exclusive, is that striatal glutamatergic levels affect total grey matter volume. A previous MRS study that examined glutamate metabolites in patients at clinical high risk for psychosis, who were mostly cannabis users, found that there were strong positive and negative correlations between thalamic glutamate levels and grey matter volume in several cortical regions^55^. These correlations were much less evident in healthy controls. One interpretation of these findings suggested by the authors was that subcortical glutamate dysfunction in psychosis drives loss of cortical grey matter volume. We, however, find this less biologically plausible for the correlation between grey matter volume and caudate Glx that we report as the associative striatum where the voxel was placed is the main afferent region for the basal ganglia receiving widespread glutamatergic projections, but cannot completely discount this possibility. A further possibility is that increased connectivity between cortical and subcortical structures with glutamate in the associative striatum drives cannabis use. In the absence of longitudinal data we cannot disentangle these possibilities.

The strengths of this study are that it represents the largest sample to have investigated this issue and that clinical measures between EPC and EPMC were well matched. Patients comprised a real-world sample recruited from 16 NHS Trusts throughout England. We included a cannabis using control arm which has been missing from several previous studies in the field^56^. We used optimised techniques to obtain volumes and absolute metabolite concentrations in native space. Since both signals were acquired in native space we did not correct for intracranial volume as this would over-correct for the relationship. ICV was matched across EPC and EPMC groups and would not explain the difference between groups. We checked to see if the same relationship existed in different tissue classes using CSF and white matter as control regions.

There are limitations of this study. The cross-sectional nature of the current investigation means it is difficult to disentangle causal relationships. It may be that the failure to find group differences is due to a Type II error with relatively modest sample sizes. Nonetheless we did power this study from pilot data, and also a study published after our pilot data also found differences between cannabis and non cannabis using patients in a smaller sample (35 patients)^26^ whereas this study is the largest such study to date. It is worth noting that powering a future study from our findings (alpha 0.05, power 80%): 140 patients (70 EPC, 70 EPMC) would be required for powering a study for the difference in Head of Caudate Glx; 680 (340 EPC, 340 EPMC) for powering a study for difference in hippocampal Glx; and 832 patients (416 EPC, 416 EPMC) to power a study in Anterior Cingulate Cortex. Hence the group differences here, if they do exist, are small and would require hundreds of patients (not including controls) to detect.

There is the ever present issue of relying upon retrospective measures to ascertain cannabis use history^56,57^. We did not collect data on the Duration of Untreated Psychosis as should be undertaken in future studies. The MRS signal does not distinguish between neuronal or metabolic pools of glutamate. Furthermore the MRS Signal to Noise Ratio (SNR) differed between EPC and EPMC groups in the caudate. We do not find this major limitation as SNR was within acceptable limits in both groups and there were no differences in other quality parameters. Differences between SNR would not explain the correlation between grey matter volume and caudate Glx seen in the EPC group. We did not measure dopaminergic activity which would be an important separate study to do.

Collectively we showed no alterations in glutamatergic indices in patients with early psychosis with and without a history of cannabis use. However, we find evidence for altered volume/striatal glutamate relationships in patients with a history of cannabis use. This adds to an accumulating array of evidence^26,29,30^ which may suggest the glutamate system as a target for therapy in the dual diagnosis group.

## Supplementary information

SUPPLEMENTARY MATERIAL
