# Supplementary material for: Association of cannabis with glutamatergic levels in patients with early psychosis: Evidence for altered volume striatal glutamate relationships in patients with a history of cannabis use in early psychosis
Source: Transl Psychiatry. 2020 Apr 21;10:111. doi: 10.1038/s41398-020-0790-1 (PMC7174331; doi:10.1038/s41398-020-0790-1)
Supplement: Supplementary file 1 — SUPPLEMENTARY MATERIAL [file 41398_2020_790_MOESM1_ESM.docx]

**SUPPLEMENTARY MATERIAL**

**Supplementary Methods:**

*MR Spectroscopy acquisition:* The anterior cingulate voxel (TR 3000ms, TE 30ms, 96 averages) was planned from axial slice 16mm superior to the genu of the corpus collosum. A 2x2x2 cm^3^ voxel was placed centrally to maximize grey matter volume. The hippocampal voxel was placed over the center of left hippocampus (TR 3000ms, TE 30ms, 96 averages) and a 2x2x1.5 cm^3^ voxel prescribed aiming to avoid amygdalar intrusion. A 2x2x2 cm^3^ voxel from left head of caudate was prescribed from an axial slice 12 mm superior to the anterior-commissure. The caudate voxel was planned in a region to cover the associative striatum with the anterior edge of voxel abutting at the edge of head of caudate and the medial edge to avoid CSF. Coronal and sagittal images were checked to include head of caudate. Image acquisition time was 6 minutes for each voxel. Unsuppressed water spectra were also acquired with 16 averages for eddy current correction and quantification. Before data acquisition the voxel was prescanned twice with target linewidths less than 7 Hertz (anterior cingulate), 11Hz (hippocampus) and 10 Hertz (caudate) after shimming.

*Spectroscopy Analysis:* LCModel Version 6.3-1L (s-provencher.com/lcmodel.shtml) was used to estimate metabolite concentrations using a standard basis set of 16 metabolites (L-alanine, aspartate, creatine, phosphocreatine, GABA, glucose, glutamine, glutamate, glycerophosphocholine, glycine, myo-inositol, L-lactate, N-acetylaspartate, N-acetylaspartylglutamate, phosphocholine, taurine). Eddy current correction was undertaken and water scaling was done to estimate absolute concentrations of metabolites using the unsuppressed reference water signal. We calculated tissue segmentation of the spectroscopy voxel using GannetRegister and GannetSegment (<http://www.gabamrs.com>) which co-registers the voxel to the anatomical T1 weighted image and uses SPM 12 to segment the voxel into fractions of grey matter (GM), white matter (WM) and cerebrospinal fluid (CSF). To correct for partial volumes, metabolite concentrations were corrected using the formula:

Raw concentration * 43300 x fraction_(GM)_ + 35880 x fraction_(WM)_ + 55556 x fraction_(CSF)_

35880 x (1-fraction_(CSF)_)

with water concentrations in mM for grey matter (43300 mM), white matter (35880 mM and CSF (55556 mM) as recommend from the LCModel manual and derived from published literature(1, 2). No corrections were performed for T_1_ or T_2_ relaxation except for assuming the T_2_ of tissue water to be 80 ms.

**Supplementary Figures & Tables:**

**sFigure 1: MR Spectroscopy Voxel acquisition**

(a) Anterior Cingulate Cortex Voxel

**
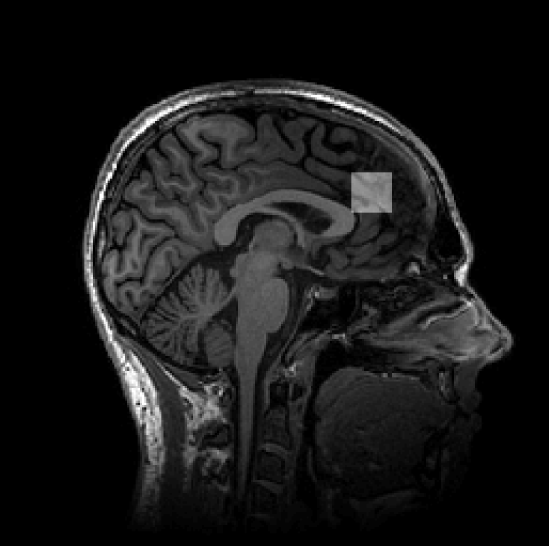

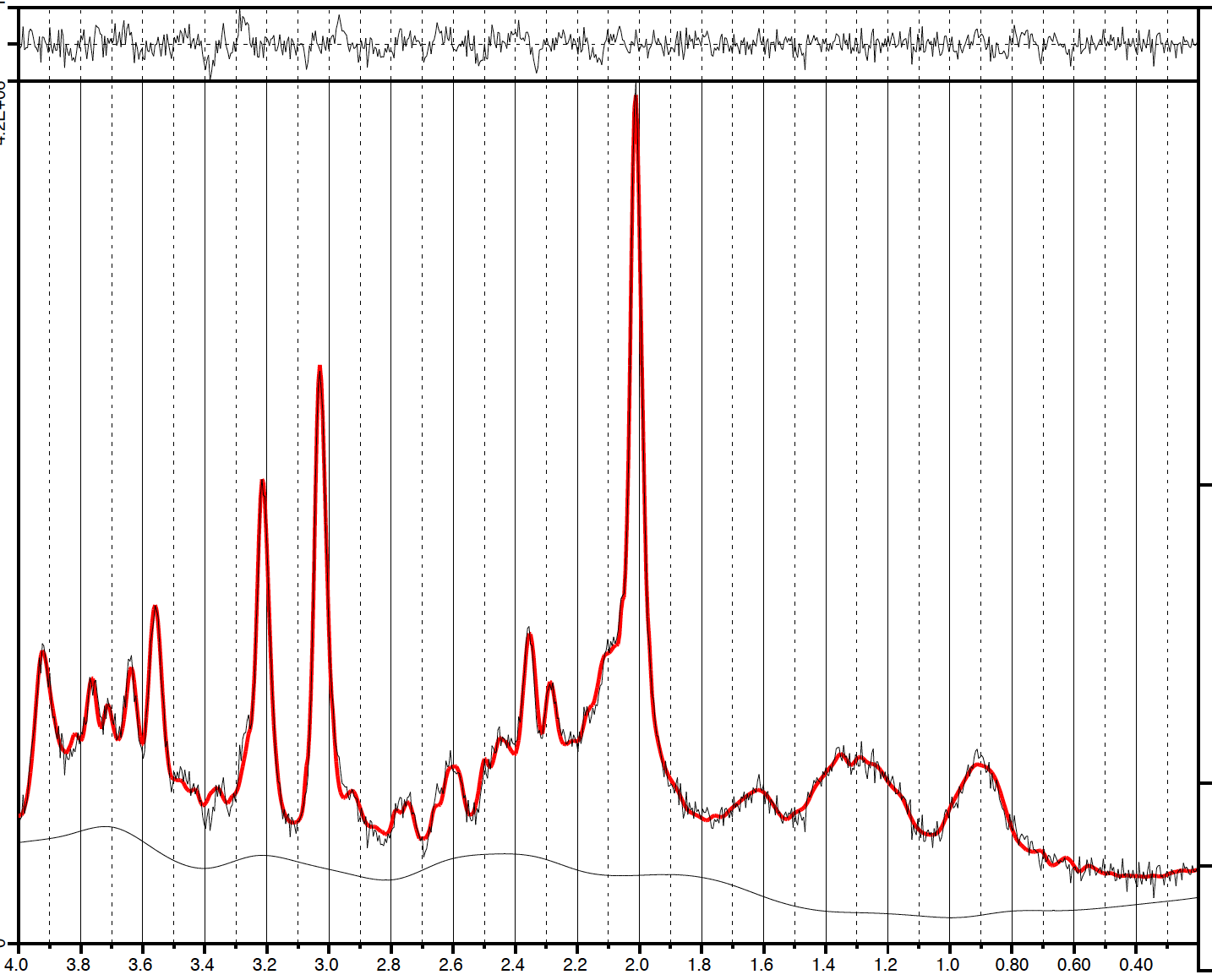
**

(b) Hippocampus Voxel


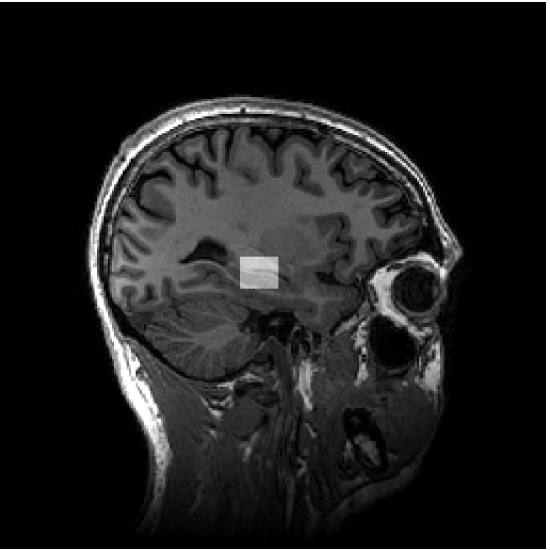

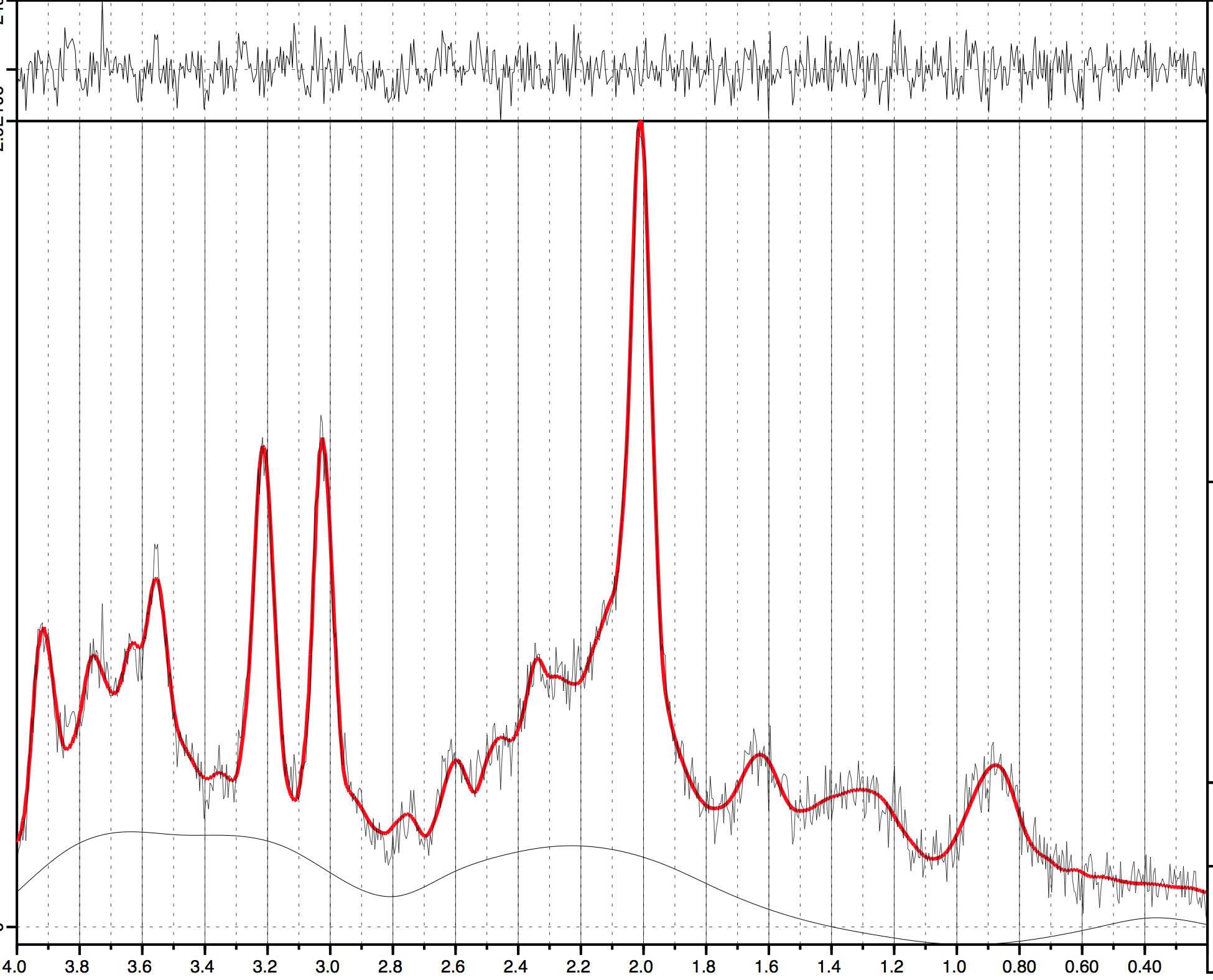


(c) Head of Caudate Voxel

**
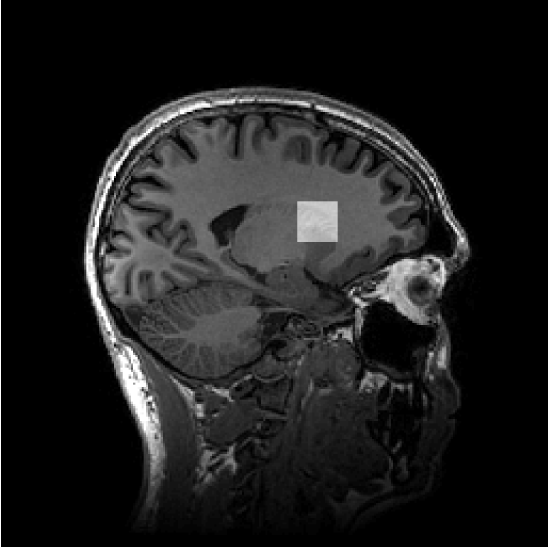

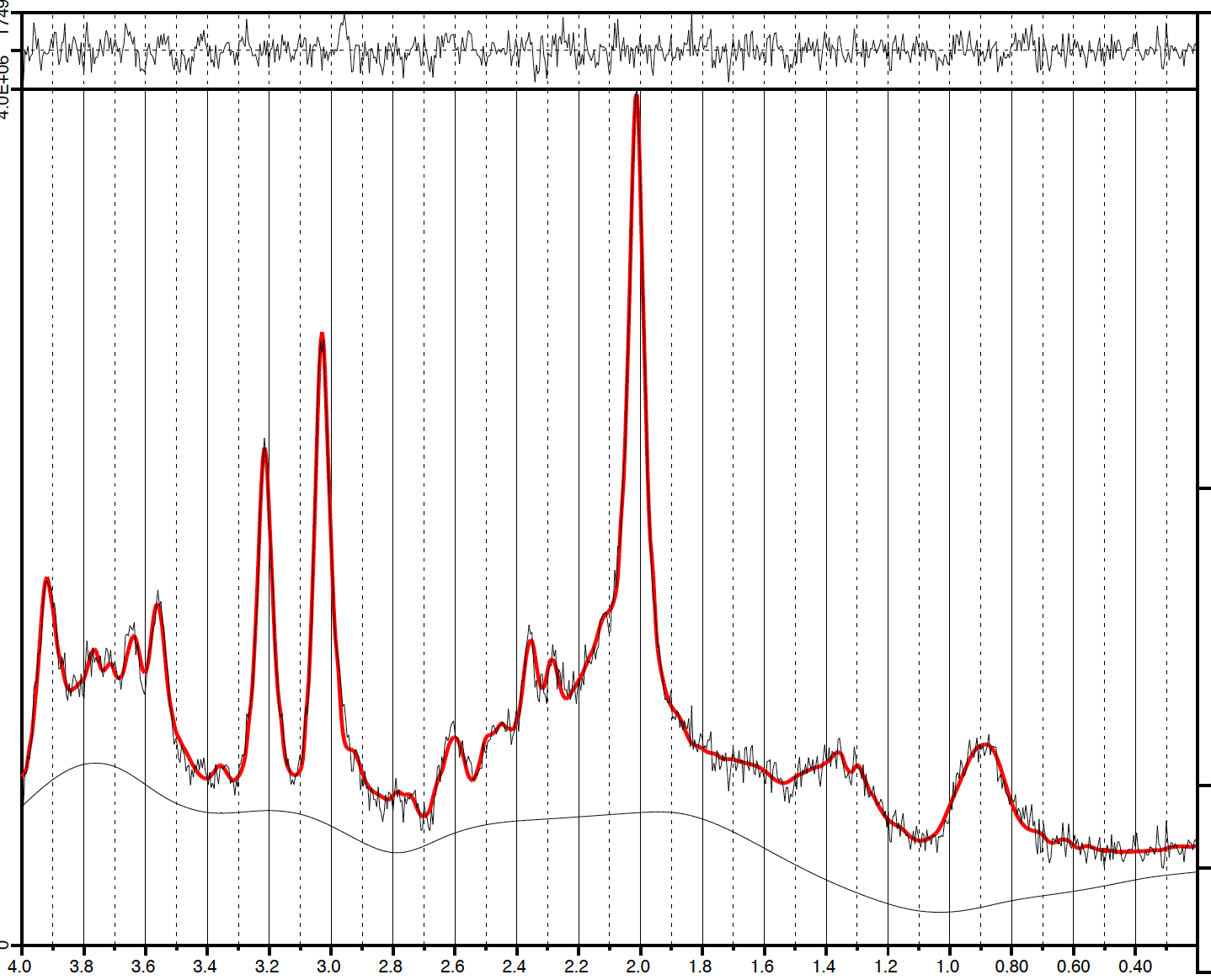
**

**sTable 1 Self reported cannabis frequency by group**

|  | EPC* | EPMC | HCC | HCMC |
| --- | --- | --- | --- | --- |
| Never use |  | 16 (64%) |  | 11 (50%) |
| Only once or twice |  | 8 (32%) |  | 9 (40.9%) |
| A few times a year |  | 1 (4%) |  | 2 (9.1%) |
| A few times a month | 4 (13.8%) |  | 3 (18.8%) |  |
| More than once a week | 7 (24.1%) |  | 4 (25.0%) |  |
| Daily | 17 (58.6%) |  | 9 (56.3%) |  |

* 1 case missing as self-report history did not tally with clinician and UDS assessment

**sTable 2a: MRS quality measures by group**

|  | **EPC** | **EPMC** | **HCC** | **HCMC** | **p value** |
| --- | --- | --- | --- | --- | --- |
| **n(ACC)** | 29 | 25 | 16 | 22 |  |
| **n(HCC)** | 29 | 25 | 16 | 22 |  |
| **n(CAU)** | 26 | 22 | 16 | 21 |  |
| **SNR(ACC)** | 32.90 (5.63) | 32.40 (5.42) | 31.50 (10.24) | 31.86 (7.85) | 0.920 |
| **SNR(HCC)** | 15.28 (4.04) | 15.12 (2.83) | 15.13 (1.93) | 16.00 (3.52) | 0.789 |
| **SNR(CAU)** | 19.19 (4.47) | 22.86 (3.90) | 20.31 (4.42) | 21.19 (5.07) | 0.046^1^ |
| **FWHM(ACC)** | 0.04 (0.01) | 0.04 (0.01) | 0.04 (0.01) | 0.04 (0.08) | 0.816 |
| **FWHM(HCC)** | 0.07 (0.02) | 0.07 (0.02) | 0.07 (0.02) | 0.07 (0.01) | 0.838 |
| **FWHM(CAU)** | 0.06 (0.01) | 0.07 (0.01) | 0.07 (0.02) | 0.07 (0.02) | 0.442 |
| **CRGlx(ACC)** | 5.72 (0.80) | 6.20 (1.04) | 6.25 (1.13) | 6.36 (1.79) | 0.246 |
| **CRGlx(HCC)** | 10.31 (2.69) | 9.76 (2.17) | 9.56 (1.41) | 10.55 (2.61) | 0.503 |
| **CRGlx(CAU)** | 9.73 (2.66) | 9.23 (3.02) | 10.19 (1.87) | 10.19 (2.62) | 0.600 |

**Legend:** Quality control measures presented with mean, standard deviation in brackets. P Values based on ANOVA across groups. EPC: Early Psychosis with Cannabis use; EPMC: Early Psychosis with minimal Cannabis use; HCC: Healthy Controls with Cannabis Use; HCMC: Healthy Controls with minimal Cannabis Use. n: number of participants. SNR: Signal to Noise ratio; FWHM: Full Width Half Maximum; CRLBGlx: Cramer-Rao Lower Bound for Glx; ACC Anterior Cingulate; HCC: Hippocampus. All Caudate measures presented after exclusion of 2 EPC cases with Cramer-Rao Lower Bound for Glx values ≥20% excluded

1 Caudate SNR EPC vs EPMC p=0.035

**sTable 2b: Voxel tissue class classification by group**

|  | **EPC** | **EPMC** | **HCC** | **HCMC** | **p value** |
| --- | --- | --- | --- | --- | --- |
| **ACC(**GM) | 0.62 (0.06) | 0.61 (0.05) | 0.63 (0.06) | 0.62 (0.04) | 0.722 |
| **ACC(**WM) | 0.07 (0.02) | 0.07 (0.02) | 0.07 (0.03) | 0.08 (0.03) | 0.618 |
| **ACC(**CSF) | 0.31 (0.06) | 0.32 (0.06) | 0.30 (0.07) | 0.31 (0.05) | 0.810 |
| **HCC(**GM) | 0.55 (0.06) | 0.56 (0.07) | 0.57 (0.07) | 0.58 (0.07) | 0.329 |
| **HCC(**WM) | 0.39 (0.08) | 0.39 (0.07) | 0.37 (0.08) | 0.36 (0.08) | 0.585 |
| **HCC(**CSF) | 0.06 (0.03) | 0.06 (0.03) | 0.06 (0.04) | 0.06 (0.02) | 0.907 |
| **CAU(**GM) | 0.51 (0.06) | 0.50 (0.06) | 0.49 (0.04) | 0.50 (0.05) | 0.762 |
| **CAU(**WM) | 0.45 (0.05) | 0.47 (0.06) | 0.47 (0.05) | 0.46 (0.05) | 0.436 |
| **CAU(**CSF) | 0.04 (0.03) | 0.03 (0.02) | 0.04 (0.04) | 0.04 (0.02) | 0.612 |

**Legend:** Date presented as proportion of total voxel, standard deviation in brackets. P Values based on ANOVA across groups. EPC: Early Psychosis with Cannabis use; EPMC: Early Psychosis with minimal Cannabis use; HCC: Healthy Controls with Cannabis Use; HCMC: Healthy Controls with minimal Cannabis Use. GM: Grey Matter; WM: White Matter; CSF: Cerebrospinal fluid. All Caudate measures presented after exclusion of 2 EPC cases with Cramer-Rao Lower Bound for Glx values ≥20% excluded

| **Further sensitivity analyses**  The reviewers asked for further data to be presented to consider (i) the impact of partial volume correction (using full correction described above; correction for CSF only *[metabolite/(1 − fraction CSF)]*; and no correction (ii) whether the correlation results held for right as well as left sided structures and (iii) whether the results also held for the Glutamate (Glu) metabolite as well as Glx (Glutmate+Glutamine) reported in the main manuscript. We present these data as exploratory sensitivity analysis below.  *A. Total Glx and Glu levels by Group with various partial volume correction methods*  There were no significant differences between groups whether Glu or Glx was used as a metabolite and whichever partial volume correction method was used  *B. Correlation between grey matter and caudate glutamatergic indices:*  Data for EPC and HCC groups is shown in sTable4 and sTable 5. Briefly these results show (i) using alternative methods of partial volume correction had no to limited effect on the main results; (ii) the correlation to volume to caudate glutamatergic indices found in the EPC group holds for both left and right sided structures and (iii) the results remain significant in the same direction, with slightly diminished correlation coefficient, when Glu is considered rather than Glx although do not remain significant for the relationship of hippocampus to Caudate Glutamate. There were trend level relationships in the HCC group in the same direction as shown below (sTable 5). There were no significant relationships in the other groups (EPMC, HCMC) and this data is not presented further.  **sTable 3: Relationship between grey matter and caudate glutamatergic indices in EPC group:**   \|  \|  \|  \| Caudate Glx (Glutamine+Glutamate) \| \| \| Caudate Glutamate* \| \| \| \| --- \| --- \| --- \| --- \| --- \| --- \| --- \| --- \| --- \| \|  \|  \|  \| No correction \| CSF correction \| Full correction \| No correction \| CSF correction \| Full correction \| \|  \| Total Grey Volume \| *r* \| 0.566 \| 0.56 \| 0.565 \| 0.473 \| 0.464 \| 0.472 \| \|  \| *p* \| 0.003 \| 0.003 \| 0.003 \| 0.013 \| 0.015 \| 0.013 \| \|  \| *n* \| 26 \| 26 \| 26 \| 27 \| 27 \| 27 \| \| Left \| Cortex \| *r* \| 0.528 \| 0.521 \| 0.526 \| 0.441 \| 0.547 \| 0.438 \| \| *p* \| 0.006 \| 0.006 \| 0.006 \| 0.021 \| 0.003 \| 0.022 \| \| *n* \| 26 \| 26 \| 26 \| 27 \| 27 \| 27 \| \| Hippocampus \| *r* \| 0.461 \| 0.502 \| 0.52 \| 0.257 \| 0.514 \| 0.331 \| \| *p* \| 0.018 \| 0.009 \| 0.006 \| 0.196 \| 0.006 \| 0.092 \| \| *n* \| 26 \| 26 \| 26 \| 27 \| 27 \| 27 \| \| Amygdala \| *r* \| 0.554 \| 0.591 \| 0.607 \| 0.375 \| 0.528 \| 0.449 \| \| *p* \| 0.003 \| 0.001 \| 0.001 \| 0.054 \| 0.005 \| 0.019 \| \| *n* \| 26 \| 26 \| 26 \| 27 \| 27 \| 27 \| \| Right \| Cortex \| *r* \| 0.602 \| 0.593 \| 0.597 \| 0.516 \| 0.624 \| 0.51 \| \| *p* \| 0.001 \| 0.001 \| 0.001 \| 0.006 \| 0.001 \| 0.007 \| \| *n* \| 26 \| 26 \| 26 \| 27 \| 27 \| 27 \| \| Hippocampus \| *r* \| 0.384 \| 0.42 \| 0.436 \| 0.206 \| 0.421 \| 0.274 \| \| *p* \| 0.053 \| 0.033 \| 0.026 \| 0.302 \| 0.033 \| 0.167 \| \| *n* \| 26 \| 26 \| 26 \| 27 \| 27 \| 27 \| \| Amygdala \| *r* \| 0.463 \| 0.492 \| 0.507 \| 0.359 \| 0.478 \| 0.412 \| \| *p* \| 0.017 \| 0.011 \| 0.008 \| 0.066 \| 0.012 \| 0.033 \| \| *n* \| 26 \| 26 \| 26 \| 27 \| 27 \| 27 \|   **sTable 4: Relationship between grey matter and caudate glutamatergic indices in HCC group:**   \|  \|  \|  \| **Caudate Glx (Glutamine+Glutamate)** \| \| \| **Caudate Glutamate*** \| \| \| \| --- \| --- \| --- \| --- \| --- \| --- \| --- \| --- \| --- \| \|  \|  \|  \| No correction \| CSF correction \| Full correction \| No correction \| CSF correction \| Full correction \| \|  \| Total Grey Volume \| *r* \| 0.485 \| 0.456 \| 0.451 \| 0.391 \| 0.371 \| 0.376 \| \|  \| *p* \| 0.057 \| 0.076 \| 0.08 \| 0.135 \| 0.157 \| 0.151 \| \|  \| *n* \| 16 \| 16 \| 16 \| 16 \| 16 \| 16 \| \| **Left** \| Cortex \| *r* \| 0.467 \| 0.437 \| 0.432 \| 0.376 \| 0.457 \| 0.361 \| \| *p* \| 0.068 \| 0.09 \| 0.095 \| 0.151 \| 0.075 \| 0.17 \| \| *n* \| 16 \| 16 \| 16 \| 16 \| 16 \| 16 \| \| Hippocampus \| *r* \| 0.47 \| 0.477 \| 0.479 \| 0.36 \| 0.477 \| 0.399 \| \| *p* \| 0.066 \| 0.062 \| 0.061 \| 0.17 \| 0.062 \| 0.126 \| \| *n* \| 16 \| 16 \| 16 \| 16 \| 16 \| 16 \| \| Amygdala \| *r* \| 0.403 \| 0.417 \| 0.423 \| 0.276 \| 0.43 \| 0.336 \| \| *p* \| 0.122 \| 0.108 \| 0.103 \| 0.3 \| 0.097 \| 0.204 \| \| *n* \| 16 \| 16 \| 16 \| 16 \| 16 \| 16 \| \| **Right** \| Cortex \| *r* \| 0.485 \| 0.455 \| 0.449 \| 0.388 \| 0.482 \| 0.374 \| \| *p* \| 0.057 \| 0.077 \| 0.081 \| 0.137 \| 0.059 \| 0.154 \| \| *n* \| 16 \| 16 \| 16 \| 16 \| 16 \| 16 \| \| Hippocampus \| *r* \| 0.374 \| 0.414 \| 0.424 \| 0.202 \| 0.417 \| 0.293 \| \| *p* \| 0.154 \| 0.11 \| 0.102 \| 0.452 \| 0.108 \| 0.27 \| \| *n* \| 16 \| 16 \| 16 \| 16 \| 16 \| 16 \| \| Amygdala \| *r* \| 0.417 \| 0.455 \| 0.469 \| 0.241 \| 0.443 \| 0.347 \| \| *p* \| 0.108 \| 0.076 \| 0.067 \| 0.368 \| 0.086 \| 0.187 \| \| *n* \| 16 \| 16 \| 16 \| 16 \| 16 \| 16 \|   **Legend:** r: Pearson’s r coefficient; p: p-Value; n: number of participants; CSF cerebrospinal fluid; red p-value indicates <0.05; green p-value indicates trend level significance 0.05<p<0.1; *Caudate Glu variables logrhythmically transformed after Shapiro-Wilks test for normality. |  |
| --- | --- | --- | --- | --- | --- | --- | --- | --- | --- | --- | --- | --- | --- | --- | --- | --- | --- | --- | --- | --- | --- | --- | --- | --- | --- | --- | --- | --- | --- | --- | --- | --- | --- | --- | --- | --- | --- | --- | --- | --- | --- | --- | --- | --- | --- | --- | --- | --- | --- | --- | --- | --- | --- | --- | --- | --- | --- | --- | --- | --- | --- | --- | --- | --- | --- | --- | --- | --- | --- | --- | --- | --- | --- | --- | --- | --- | --- | --- | --- | --- | --- | --- | --- | --- | --- | --- | --- | --- | --- | --- | --- | --- | --- | --- | --- | --- | --- | --- | --- | --- | --- | --- | --- | --- | --- | --- | --- | --- | --- | --- | --- | --- | --- | --- | --- | --- | --- | --- | --- | --- | --- | --- | --- | --- | --- | --- | --- | --- | --- | --- | --- | --- | --- | --- | --- | --- | --- | --- | --- | --- | --- | --- | --- | --- | --- | --- | --- | --- | --- | --- | --- | --- | --- | --- | --- | --- | --- | --- | --- | --- | --- | --- | --- | --- | --- | --- | --- | --- | --- | --- | --- | --- | --- | --- | --- | --- | --- | --- | --- | --- | --- | --- | --- | --- | --- | --- | --- | --- | --- | --- | --- | --- | --- | --- | --- | --- | --- | --- | --- | --- | --- | --- | --- | --- | --- | --- | --- | --- | --- | --- | --- | --- | --- | --- | --- | --- | --- | --- | --- | --- | --- | --- | --- | --- | --- | --- | --- | --- | --- | --- | --- | --- | --- | --- | --- | --- | --- | --- | --- | --- | --- | --- | --- | --- | --- | --- | --- | --- | --- | --- | --- | --- | --- | --- | --- | --- | --- | --- | --- | --- | --- | --- | --- | --- | --- | --- | --- | --- | --- | --- | --- | --- | --- | --- | --- | --- | --- | --- | --- | --- | --- | --- | --- | --- | --- | --- | --- | --- | --- | --- | --- | --- | --- | --- | --- | --- | --- | --- | --- | --- | --- | --- | --- | --- | --- | --- | --- | --- | --- | --- | --- | --- | --- | --- | --- | --- | --- | --- | --- | --- | --- | --- | --- | --- | --- | --- | --- | --- | --- | --- | --- | --- | --- | --- | --- | --- | --- | --- | --- | --- | --- | --- | --- | --- | --- | --- | --- | --- | --- | --- | --- | --- | --- | --- | --- |

**sTable 5 Mean Metabolite concentrations across groups**

|  | **EPC** | | **EPMC** | | **HCC** | | **HCMC** | |  |  |
| --- | --- | --- | --- | --- | --- | --- | --- | --- | --- | --- |
|  | x̅ | SD | x̅ | SD | x̅ | SD | x̅ | SD | p Value |  |
| ACC Cr | 12.31 | 1.34 | 12.36 | 1.14 | 11.81 | 1.47 | 12.40 | 0.91 | 0.468 |  |
| ACC Glu | 16.61 | 2.20 | 16.21 | 2.30 | 16.10 | 1.79 | 16.24 | 1.81 | 0.568 |  |
| ACC MI | 9.30 | 1.30 | 9.44 | 1.18 | 9.20 | 1.42 | 9.21 | 0.89 | 0.951 |  |
| ACC NAA | 15.39 | 1.63 | 15.36 | 1.97 | 15.31 | 2.31 | 15.76 | 1.32 | 0.596 |  |
| ACC GPCplusPCH | 3.14 | 0.66 | 3.19 | 0.50 | 2.95 | 0.42 | 3.06 | 0.38 | 0.664 |  |
| HCC Cr | 7.58 | 1.00 | 7.43 | 1.11 | 7.48 | 0.96 | 7.46 | 0.88 | 0.926 |  |
| HCC Glu | 8.15 | 1.21 | 8.07 | 1.59 | 8.04 | 1.28 | 8.00 | 1.35 | 0.959 |  |
| HCC MI | 6.25 | 1.26 | 6.53 | 1.17 | 6.82 | 1.34 | 6.55 | 1.16 | 0.479 |  |
| HCC NAA | 9.55 | 1.19 | 9.21 | 1.04 | 9.12 | 1.06 | 9.21 | 1.13 | 0.464 |  |
| HCC GPCplusPCH | 2.51 | 0.37 | 2.52 | 0.41 | 2.40 | 0.32 | 2.50 | 0.39 | 0.592 |  |
| CAU Cr | 8.26 | 1.26 | 8.21 | 1.22 | 7.84 | 1.07 | 8.31 | 1.09 | 0.632 |  |
| CAU Glu | 8.01 | 1.94 | 8.75 | 1.89 | 7.92 | 1.99 | 7.89 | 1.03 | 0.34 |  |
| CAU MI | 4.33 | 1.64 | 4.40 | 1.19 | 3.92 | 2.31 | 4.57 | 1.24 | 0.677 |  |
| CAU NAA | 9.93 | 1.17 | 10.06 | 0.96 | 9.47 | 1.14 | 9.78 | 1.31 | 0.449 |  |
| CAU GPCplusPCH | 1.97 | 0.38 | 1.97 | 0.32 | 1.72 | 0.40 | 1.94 | 0.39 | 0.144 |  |
|  |  |  |  |  |  |  |  |  |  |  |
| **Legend:** Mean metabolite values and standard deviations after correction for partial volume effects. All p Values based on ANOVA across groups. EPC: Early Psychosis with Cannabis use; EPMC: Early Psychosis without Cannabis use; HCC: Healthy Controls with Cannabis Use; HCMC: Healthy Controls without Cannabis. ACC: Anterior Cingulate Cortex; HCC: Hippocampus; CAU: caudate. Cr: Creatine; Glu: Glutamate; MI myo-inositol; NAA: N-acetylaspartate; GPCplusPCH: glycerophosphocholine+phosphocholine. | | | | | | | | | |  |

**References**

1. Kreis R, Ernst T, Ross BD (1993): Development of the human brain: In vivo quantification of metabolite and water content with proton magnetic resonance spectroscopy. *Magn Reson Med*. 30: 424–437.

2. Gasparovic C, Song T, Devier D, Bockholt HJ, Caprihan A, Mullins PG, *et al.* (2006): Use of tissue water as a concentration reference for proton spectroscopic imaging. *Magn Reson Med*. 55: 1219–1226.
